# Clinical Significance of MicroRNAs in Patients with Sepsis: Protocol for a Systematic Review and Meta-Analysis

**DOI:** 10.3390/diagnostics9040211

**Published:** 2019-12-03

**Authors:** Daisuke Hasegawa, Kazuma Yamakawa, Kohei Taniguchi, Shuhei Murao, Osamu Nishida

**Affiliations:** 1 Department of Anesthesiology and Critical Care Medicine, Fujita Health University School of Medicine, 1-98 Dengakugakubo, Kutsukake-cho, Toyoake 470-1192, Japan; hasegawa.daisuke.0407@gmail.com (D.H.); nishida@fujita-hu.ac.jp (O.N.); 2 Division of Trauma and Surgical Critical Care, Osaka General Medical Center, 3-1-56 Bandai-Higashi, Sumiyoshi, Osaka 558-8558, Japan; shmu20268271@gmail.com; 3 Department of General and Gastroenterological Surgery, Osaka Medical College, 2-7 Daigaku-machi, Takatsuki, Osaka 569-8686, Japan; sur144@osaka-med.ac.jp; 4 Translational Research Program, Osaka Medical College, 2-7 Daigaku-machi, Takatsuki, Osaka 569-8686, Japan

**Keywords:** sepsis, microRNA, diagnosis, prognosis, meta-analysis

## Abstract

Sepsis is a dysregulated immune response that leads to organ dysfunction and has high mortality rates despite recent therapeutic advancements. Accurate diagnosis and risk stratification are important for effective sepsis treatment; however, no decisive diagnostic or prognostic biomarkers are currently available. To understand whether microRNA (miRNA) might be useful biomarkers of sepsis, we aim to assess the diagnostic and prognostic accuracy of three miRNAs (122, 150, and 223) in sepsis patients via a meta-analysis of relevant published data. We will search electronic bibliographic databases (MEDLINE, EMBASE, and the Cochrane Central Register of Controlled Trials) for pertinent retrospective and prospective studies in October 2019. Two reviewers will evaluate the collected titles, abstracts, and full articles, and extract the data. We will assess the included studies using the Quality Assessment of Diagnostic Accuracy Studies-2 tool. If feasible, we will use bivariate random effects and hierarchical summary receiver operating characteristic (ROC) models to estimate summary ROCs, pooled sensitivity and specificity values, and the corresponding 95% confidence intervals. We will evaluate heterogeneity via clinical and methodological subgroup and sensitivity analyses. This systematic review will clarify the diagnostic and prognostic accuracy of select miRNAs in sepsis. It may also identify knowledge gaps in sepsis’ diagnosis and prognosis.

## 1. Introduction

Sepsis is a life-threatening condition in which a dysregulated systemic host response to microbial pathogens causes a disproportionate inflammatory response and multi-organ failure [1]. Although mortality rates for sepsis have decreased dramatically owing to recent advancements in bundled care, they are still relatively high [2]. Moreover, sepsis places a tremendous financial burden on health care systems in terms of diagnostic and treatment costs [3]. The appropriate treatment of septic patients that avoids unnecessary procedures, reduces costs, and improves mortality rates requires accurate diagnosis and risk stratification. Although various laboratory biomarkers are available for these purposes, none are used universally because their accuracy is limited.

MicroRNAs (miRNAs) are short (20–25 nucleotides), evolutionarily conserved, single-stranded, non-coding RNAs that bind to complementary sites in the 3′ untranslated regions of messenger RNAs to repress their transcription or induce their degradation [4]. MiRNAs have been associated with the development of various human diseases, such as cardiovascular, autoimmune, and malignant disorders [5,6,7], and have gradually been recognized as non-invasive, sufficiently sensitive tools for disease detection and prognosis.

It is now apparent that miRNA dysregulation (upregulation or downregulation) contributes to the development and progression of sepsis [8]. miRNA dysregulation has been reported in patients with sepsis [9], and numerous studies have identified potential miRNA biomarkers for sepsis. Most notably, a number of articles have reported the association between miRNA-122, -150, and -223 [10] and sepsis diagnosis or prognostication. However, a meta-analysis assessing the usefulness of miRNAs for sepsis diagnosis and prognosis is yet to be performed. A thorough systematic review and meta-analysis of data gathered from a broad set of published articles will allow us to comprehensively evaluate the diagnostic and prognostic potential of specific miRNAs in septic patients.

The objective of the study here presented is to assess the diagnostic and prognostic performance of miRNA-122, -150, and -223 in critically ill and septic patients, via a meta-analysis of published data.

## 2. Methods and Analysis

### 2.1. Protocol

**Figure 1:**
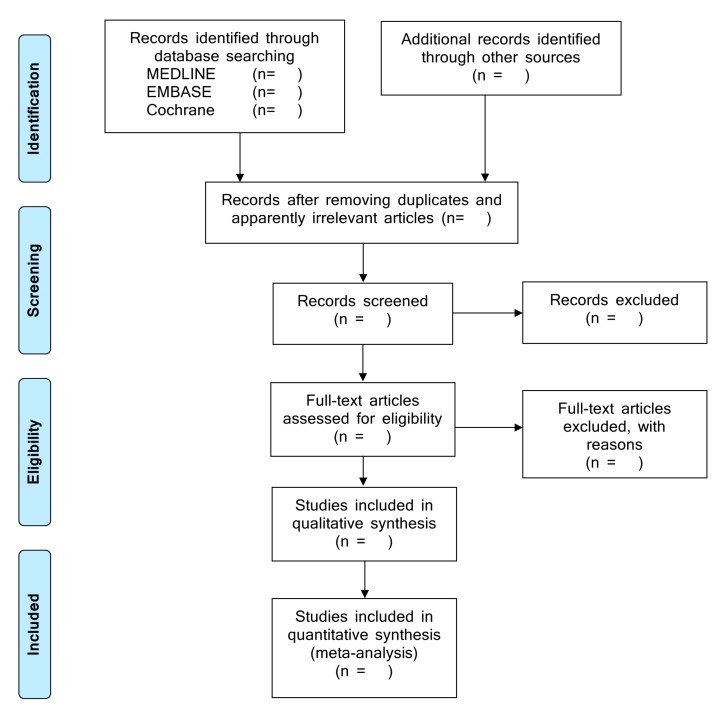
Preferred Reporting Items for Systematic Reviews and Meta-Analyses (PRISMA) flow chart.

This study will follow the guidance on systematic reviews and meta-analyses provided by the Preferred Reporting Items for Systematic Reviews and Meta-Analyses (PRISMA) [11,12,13] and Meta-Analysis of Observational Studies in Epidemiology [14] guidelines, in addition to the recommendations of the Cochrane Diagnostic Test Accuracy Working Group [15]. This study’s protocol is now in the process of being registered in PROSPERO, an International Prospective Register of Systematic Reviews (http://www.crd.york.ac.uk/PROSPERO/). Figure 1 shows the PRISMA flow chart of this study.

### 2.2. Focused Review Question

The question we will asked is whether miRNA-122, -150, and -223 are valid biomarkers for diagnosing sepsis in critically ill patients and stratifying mortality risks in septic patients.

### 2.3. Types of Studies

Randomized controlled trials, cohort studies, case-control studies, and cross-sectional studies will be included. The included studies should hold enough information for a 2 × 2 contingency table (true and false, positive and negative). We will exclude animal studies, as well as studies predominantly comprising post-cardiac surgery, perioperative patients, or patients with heart failure. We will also exclude narrative reviews, correspondence, case reports, expert opinions, and editorials.

### 2.4. Types of Participants

We will include studies that compare the levels of miRNA-122, -150, and -223 between: (1) patients with sepsis and patients with critical illnesses, (2) patients with sepsis and healthy volunteers (controls), and (3) patients with sepsis who had died and patients with sepsis who had survived. Since the definition of “critical illness” is ambiguous, we will include only critical illnesses with universally accepted definitions, such as systemic inflammatory response syndrome (SIRS), sepsis, and acute respiratory distress syndrome. Critically ill patients are eligible for this study if they have been treated in emergency rooms, hospital wards, and intensive care units.

### 2.5. Studied Tests

We will include studies that specify the index test used for the measurement of miRNA-122, -150, or -223 levels in plasma or serum.

### 2.6. Reference Standards

Studies that used one of the three reference gold standards (Sepsis-1, -2, and -3) for sepsis [1,16,17] will be included. We will also use other explicit author-defined reference standards for sepsis, if found. We realize that the clinical diagnostic criteria for sepsis have been modified over time and differ from country to country. Articles in which the clinical diagnosis does not meet all the criteria for sepsis will be included in our review only if the authors of the articles can justify their diagnosis.

### 2.7. Exclusion Criteria

We will exclude studies in which true positive, false positive, and negative rates are not reported, cannot be calculated from the main text or supplemental files or be obtained from the authors, contain abstracts with data insufficient to assess methodological quality, or include subcohorts or duplicate cohorts previously published elsewhere.

### 2.8. Search Strategy

Search for associated articles, performed in October 2019, will include the following databases: MEDLINE (via PubMed), EMBASE, and the Cochrane Central Register of Controlled Trials. The detailed MEDLINE search strategy is shown in Table 1; the same search strategy will be used with the other two databases.

The search will extend from 2001 to the present, because the first article on miRNA was published in 2001. We will not make use of a diagnostic accuracy search filter since doing so often excludes relevant articles previously included in systematic reviews of diagnostic accuracy studies. There will be no language limitations. We will peruse the reference lists of all retrieved papers to identify additional relevant studies. We will also get in contact with the authors of ongoing or unpublished studies to obtain information about these studies.

### 2.9. Citation Management and Screening

Citations will be tracked in EndNote software (Thomson Reuters, Toronto, ON, Canada), and duplicates will be excluded using the same software. Two authors (DH and SM) will independently screen the articles by title and abstract and remove the articles that do not meet the eligibility criteria. They will resolve any disagreements via discussion, with the participation of a third author (KY), if necessary. After the initial screening, the same two authors (DH and SM) will independently review the full text of the selected studies to determine whether they should be included in the final study. Disagreements will be resolved as described above. We will follow the PRISMA flow diagram to document the study selection process.

### 2.10. Data Abstraction

Two authors (DH and SM) will extract data on the study characteristics from each included article. Data extraction sheets will include variables required to assess study quality and between-study heterogeneity. The same authors will be responsible for transferring the information and converting the data into our study-specific format, as required. A third author (KY) will arbitrate any disagreements. We will prepare 2 × 2 tables to cross-tabulate numeric data (positive or negative) from the index tests with the target disorder and will present the tabulated results in additional tables. If relevant data are missing, we will ask the authors to provide them.

### 2.11. Assessment of Risk of Bias

Two authors (DH and SM) will independently evaluate the quality of the included studies and a third (KY) will verify it, if needed. The Quality Assessment of Diagnostic Accuracy Studies-2 (QUADAS-2) tool will be used for the evaluation [18]. We will specifically assess the presence of risk of bias in patient selection, index test, reference standard, and flow and timing. For each domain, there are several signaling questions, and if the answers to all signaling questions for a domain are “yes,” we will designate the risk of bias as low. Otherwise, we will designate the risk of bias as high. If insufficient detail is reported to assess the risk of bias, we will ask the study authors for clarification. If the detail is unaffordable, we will designate the risk of bias as unclear.

### 2.12. Data Synthesis

To evaluate inter-study variability visually, we will generate forest plots and receiver operating characteristic (ROC) curves after plotting the estimated sensitivities and specificities (with 95% confidence intervals). We will use Review Manager (RevMan version 5.3, the Nordic Cochrane Centre, the Cochrane Collaboration, Copenhagen, Denmark) to report the descriptive analyses. We will pool studies only if (1) a similar threshold is used in each study, (2) the studies were performed in identical or similar settings, and (3) the studies show sufficient clinical homogeneity.

In meta-analysis, we will make use of a bivariable random effects model to fit a summary ROC curve. We will estimate indices of accuracy such as sensitivity, specificity, and likelihood ratio using the MIDAS module in the STATA (version 14.0) software program (Stata Corporation, College Station, TX, USA). We will also estimate positive predictive values, which are more clinically useful. The 95% confidence ellipse and prediction region around the averaged accuracy estimates in the ROC space will be plotted. We will create a nomogram, which is a user-friendly graphical depiction of positive and negative predictive values according to the prevalence.

### 2.13. Assessment of Heterogeneity

To examine heterogeneity, we will visually inspect the forest plots of each study’s reported sensitivity and specificity as well as the corresponding ROC curves. Statistical heterogeneity will be rigorously evaluated by analyzing the forest plots of the study estimates and, more rigorously, by using the χ^2^ test (*p* < 0.1 = significant heterogeneity) and the I^2^ statistic (I^2^ > 50% = significant heterogeneity).

### 2.14. Assessment of Publication Bias

If the number of included studies is adequate, we will assess publication bias with the Deek’s funnel plot. However, we will use this test with caution, as it lacks statistical power, which is a significant limitation. This limitation notwithstanding, there are currently no alternative methods of detecting publication bias in systematic reviews of diagnostic and prognostic test accuracy.

### 2.15. Sensitivity and Subgroup Analysis

We will perform sensitivity analyses to evaluate the robustness of the meta-analysis and exclude studies that may bias the results using the QUADAS-2 tool. If an adequate number of studies is available, we will conduct subgroup analyses to identify sources of heterogeneity. The following covariates will be included in univariate meta-regression and subgroup analyses: country, year of study publication, sepsis prevalence (<50% or ≥50%), sample size (<100 or ≥100), study setting (hospital ward, emergency room, intensive care unit, or mixed), admission category (medical or surgical), source of infection, severity of the disease (sepsis, severe sepsis, or septic shock), clinical diagnostic criteria (International Consensus Definition for sepsis from 1991, 2001, or 2016, or author-defined), and causal pathogen of sepsis (bacterial, fungal, viral, or other). We will also use the information on cutoff from each study to assess the threshold effect on heterogeneity and determine an optimal cutoff by maximizing the Youden index.

## 3. Discussion

Several recent studies have identified miRNA-122, -150, and -223 as potentially powerful diagnostic and predictive biomarkers for sepsis [10]. MiRNA-122 is overexpressed in patients with liver injuries [19], and its association with sepsis and sepsis outcomes has been described [20]. MiRNA-150 is a key regulator of immune cell differentiation and activation [21]. Its expression level correlates with the survival of septic patients [22] and discriminates between patients with SIRS and those with sepsis [23]. MiRNA-223 is thought to play a key role in hematopoietic lineage differentiation [24], and its levels are lower in septic patients than in SIRS patients and healthy individuals [25].

Despite reports attesting to the diagnostic and prognostic accuracy of miRNA-122, -150, and -223 in septic patients, to our knowledge, there are currently no systematic reviews or meta-analyses on this topic. We believe that a comprehensive, systematic, and appropriately controlled meta-analysis of all relevant currently available data will greatly further our understanding of the diagnostic and prognostic accuracy of these miRNAs in sepsis. In vitro [26] and in vivo [19,20,21,22,23,24,25] studies have already identified numerous miRNA targets, which hopefully will spur research in this area, particularly in the clinical field. We expect that our proposed study will build upon existing information, while simultaneously acting as a guide for future studies assessing the diagnostic and prognostic value of miRNAs in sepsis.

## Figures and Tables

**Table 1:** MEDLINE (Ovid) search strategy.

#1	Systemic inflammatory response syndrome[mesh] OR Systemic inflammatory response syndrome[tiab] OR SIRS[tiab]
#2	Multiple Organ Failure[mesh] OR Multiple Organ Failure[tiab] OR MOF[tiab]
#3	Sepsis[mesh] OR Sepsis[tiab] OR Septic[tiab]
#4	#1 OR #2 OR #3
#5	MicroRNAs[Mesh] OR microRNA*[tiab] OR miRNA*[tiab] OR miR*[tiab]
#6	#4 AND #5
#7	animals[mh] NOT humans[mh]
#8	#6 NOT #7

## References

[1] Singer M., Deutschman C.S., Seymour C.W., Shankar-Hari M., Annane D., Bauer M., Bellomo R., Bernard G.R., Chiche J.D., Coopersmith C.M. (2016). The Third International Consensus Definitions for Sepsis and Septic Shock (Sepsis-3). JAMA.

[2] Jawad I., Lukšić I., Rafnsson S.B. (2012). Assessing available information on the burden of sepsis: Global estimates of incidence, prevalence and mortality. J. Glob. Health.

[3] Calvert J., Hoffman J., Barton C., Shimabukuro D., Ries M., Chettipally U., Kerem Y., Jay M., Mataraso S., Das R. (2017). Cost and mortality impact of an algorithm-driven sepsis prediction system. J. Med. Econ..

[4] Taniguchi K., Sugito N., Kumazaki M., Shinohara H., Yamada N., Nakagawa Y., Ito Y., Otsuki Y., Uno B., Uchiyama K. (2015). MicroRNA-124 inhibits cancer cell growth through PTB1/PKM1/PKM2 feedback cascade in colorectal cancer. Cancer Lett..

[5] Ardekani A.M., Naeini M.M. (2010). The Role of MicroRNAs in Human Diseases. Avicenna J. Med. Biotechnol..

[6] Reid G., Kirschner M.B., van Zandwijk N. (2011). Circulating microRNAs: Association with disease and potential use as biomarkers. Crit. Rev. Oncol. Hematol..

[7] Haider B.A., Baras A.S., McCall M.N., Hertel J.A., Cornish T.C., Halushka M.K. (2014). A critical evaluation of microRNA biomarkers in non-neoplastic disease. PLoS ONE.

[8] Kingsley S.M.K., Bhat B.V. (2017). Role of microRNAs in sepsis. Inflamm. Res..

[9] Benz F., Roy S., Trautwein C., Roderburg C., Luedde T. (2016). Circulating MicroRNAs as Biomarkers for Sepsis. Int. J. Mol. Sci..

[10] Rahmel T., Schäfer S.T., Frey U.H., Adamzik M., Peters J. (2018). Increased circulating microRNA-122 is a biomarker for discrimination and risk stratification in patients defined by sepsis-3 criteria. PLoS ONE.

[11] Moher D., Liberati A., Tetzlaff J., Altman D.G., PRISMA Group (2009). Preferred reporting items for systematic reviews and meta-analyses: The PRISMA Statement. Open Med..

[12] Liberati A., Altman D.G., Tetzlaff J., Mulrow C., Gøtzsche P.C., Ioannidis J.P., Clarke M., Devereaux P.J., Kleijnen J., Moher D. (2009). The PRISMA statement for reporting systematic reviews and meta-analyses of studies that evaluate health care interventions: Explanation and elaboration. J. Clin. Epidemiol..

[13] Moher D., Shamseer L., Clarke M., Ghersi D., Liberati A., Petticrew M., Shekelle P., Stewart L.A., PRISMA-P Group (2015). Preferred reporting items for systematic review and meta-analysis protocols (PRISMA-P) 2015 statement. Syst. Rev..

[14] Stroup D.F., Berlin J.A., Morton S.C., Olkin I., Williamson G.D., Rennie D., Moher D., Becker B.J., Sipe T.A., Thacker S.B. (2000). Meta-analysis of observational studies in epidemiology: A proposal for reporting. Meta-analysis Of Observational Studies in Epidemiology (MOOSE) group. JAMA.

[15] Leeflang M.M., Deeks J.J., Gatsonis C., Bossuyt P.M. (2008). Cochrane Diagnostic Test Accuracy Working Group. Systematic reviews of diagnostic test accuracy. Ann. Intern. Med..

[16] Bone R.C., Balk R.A., Cerra F.B., Dellinger R.P., Fein A.M., Knaus W.A., Schein R.M., Sibbald W.J. (1992). Definitions for sepsis and organ failure and guidelines for the use of innovative therapies in sepsis. The ACCP/SCCM Consensus Conference Committee. American College of Chest Physicians/Society of Critical Care Medicine. Chest.

[17] Levy M.M., Fink M.P., Marshall J.C., Abraham E., Angus D., Cook D., Cohen J., Opal S.M., Vincent J.L., Ramsay G. (2003). SCCM/ESICM/ACCP/ATS/SIS. 2001 SCCM/ESICM/ACCP/ATS/SIS International Sepsis Definitions Conference. Crit. Care Med..

[18] Whiting P.F., Rutjes A.W., Westwood M.E., Mallett S., Deeks J.J., Reitsma J.B., Leeflang M.M., Sterne J.A., Bossuyt P.M., QUADAS–2 Group (2011). QUADAS–2: A revised tool for the quality assessment of diagnostic accuracy studies. Ann. Intern. Med..

[19] Roderburg C., Benz F., Vargas Cardenas D., Koch A., Janssen J., Vucur M., Gautheron J., Schneider A.T., Koppe C., Kreggenwinkel K. (2015). Elevated miR-122 serum levels are an independent marker of liver injury in inflammatory diseases. Liver Int..

[20] Wang H., Zhang P., Chen W., Feng D., Jia Y., Xie L. (2012). Serum microRNA signatures identified by Solexa sequencing predict sepsis patients’ mortality: A prospective observational study. PLoS ONE.

[21] Tsitsiou E., Lindsay M.A. (2009). MicroRNAs and the immune response. Curr. Opin. Pharmacol..

[22] Roderburg C., Luedde M., Vargas Cardenas D., Vucur M., Scholten D., Frey N., Koch A., Trautwein C., Tacke F., Luedde T. (2013). Circulating microRNA-150 serum levels predict survival in patients with critical illness and sepsis. PLoS ONE.

[23] Ma Y., Vilanova D., Atalar K., Delfour O., Edgeworth J., Ostermann M., Hernandez-Fuentes M., Razafimahatratra S., Michot B., Persing D.H. (2013). Genome-wide sequencing of cellular microRNAs identifies a combinatorial expression signature diagnostic of sepsis. PLoS ONE.

[24] Haneklaus M., Gerlic M., O’Neill L.A., Masters S.L. (2013). miR-223: Infection, inflammation and cancer. J. Intern. Med..

[25] Wang J.F., Yu M.L., Yu G., Bian J.J., Deng X.M., Wan X.J., Zhu K.M. (2010). Serum miR-146a and miR-223 as potential new biomarkers for sepsis. Biochem. Biophys. Res. Commun..

[26] Pfeiffer D., Rossmanith E., Lang I., Falkenhagen D. (2017). miR-146a, miR-146b, and miR-155 increase expression of IL-6 and IL-8 and support HSP10 in an in vitro sepsis model. PLoS ONE.

